# Mechanism-Driven Diagnostic Development: A Specimen-Aware Framework Illustrated by Colorectal Cancer and Solid Tumours

**DOI:** 10.3390/cancers18172766

**Published:** 2026-08-26

**Authors:** Ian Daniels, Andrew J. Page, Daniel Wise

**Affiliations:** 1 Origin Sciences and Granta Park, Cambridge CB21 6AD, Cambridgeshire, UK; ian.daniels@originsciences.com (I.D.); andrew.page@originsciences.com (A.J.P.); 2 Royal Devon University Healthcare NHS Foundation Trust, Barrack Road, Exeter EX53BJ, Devon, UK

**Keywords:** early detection, liquid biopsy, precision medicine, biomarkers, colorectal cancer, translational oncology, multiomics, cell-free DNA, microbiome, minimal residual disease, mechanism-driven diagnostics, biospecimens

## Abstract

Translational oncology has moved rapidly from histopathology and single-analyte biomarkers toward multi-dimensional molecular profiling. Yet many clinically deployed tests still use reductionist biomarker strategies that under-represent cancer complexity. This review examines whether a mechanistic, multi-layered, and specimen-aware approach can improve cancer detection, classification, prognosis, minimal residual disease (MRD) assessment, and therapeutic selection. Evidence across solid tumours shows that genomic alterations alone incompletely explain tumour state, metastatic behaviour, immune evasion, or therapeutic vulnerability. Integrated genome and transcriptome analyses, proteogenomics, single-cell atlases, fragmentomic, methylation based cell-free DNA assays, metabolomics and microbiome assessments reveal clinically relevant biology that single modality tests cannot determine. Minimally invasive collected specimens can extend access to screening, diagnosis and longitudinal monitoring, but the choice of specimen should be matched to disease biology and analytes that represent mechanisms of oncogenesis. However, translation remains constrained by pre-analytical variability, contamination, differences in tumour shedding behaviour, clonal haematopoiesis, translation of generated models, incomplete external validation and uncertain downstream clinical utility for emerging platforms. This review provides a commentary on the future of cancer diagnostics, the considerations and barriers to clinical translation, the relationship between utility and dimensionality of biomarkers assessed and the emerging rationale towards mechanistically grounded integrated models.

## Simple Summary

Cancer diagnostics have traditionally relied on radiological imaging and histopathological assessment of biopsied tissue, but modern translational oncology increasingly uses blood, urine, stool, saliva, cerebrospinal fluid, and minimally invasive tissue sampling to detect and characterise disease. Currently available tests still depend on a limited number of biomarkers in one biological layer, such as DNA mutations or protein abundance. Although broadly adopted across clinical practice, this reductive strategy does not consider the inter-play of molecular, cellular, immune, metabolic, and microbial regulation that shapes oncogenesis and tumour behaviour. In this review, we evaluate recent advancements in cancer biology, technology and how these combinations can and are being utilized for prospective diagnostic applications. We rationalise that cancer profiling should be “mechanistically-driven and specimen-aware.” and propose a conceptual framework that critically evaluates the benefits of using the clinical application as the driver for biological specimen selection, combined with multimodal understanding of oncogenesis to determine biomarker selection strategies. We discuss how multi-omics, single-cell and spatial methods, liquid biopsy, and artificial intelligence (AI) are enabling more informative disease models, and we examine how these advances can improve screening, diagnosis, primary treatment selection, residual disease detection, and monitoring of ongoing outcomes. We also address the practical barriers to translation, including pre-analytical variability, assay standardisation, contamination, model validation, and health-system implementation.

## 1. Introduction

Historically, cancer diagnosis has been anchored in radiological imaging and the histopathological assessment of directly biopsied or excised tissue, which for colorectal cancer (CRC) involves colonoscopic biopsy and radiological staging to direct treatment. That paradigm remains indispensable, but it is increasingly complemented by circulating tumour DNA (ctDNA), circulating tumour cells, cell-free RNA (cfRNA), protein and metabolite profiling, as part of minimally invasive testing strategies [1,2,3,4,5,6]. Liquid biopsy has already entered clinical practice for selected advanced cancers, particularly as a source of companion diagnostic information (such as treatment guidance), but the field’s larger translational ambition is broader, encapsulating early detection, dynamic treatment monitoring, residual disease assessments, and biologically informed patient stratification across healthcare pathways [7,8].

The barrier to achieving these goals is that translation has often advanced by selecting the most deployable signal rather than the most biologically informative one. A single mutation, a single methylation signature, or a single protein can be clinically informative, but cancer is not a single-layered biological phenomenon. Tumour initiation, clonal selection, immune escape, metastatic potential, and therapy response arise from interactions among genomic drivers, epigenetic modifications, transcriptional expression, proteomic dysregulation, metabolic flux and the microbial ecosystem [9,10,11,12,13]. Integrated genome, transcriptome and proteogenomic studies in CRC illustrate this clearly, the same disease class can contain distinct molecular subtypes, prognostic signatures, and pathway dependencies that are not discernible from one “-omic layer” alone [9,10,14].

**Figure 1:**
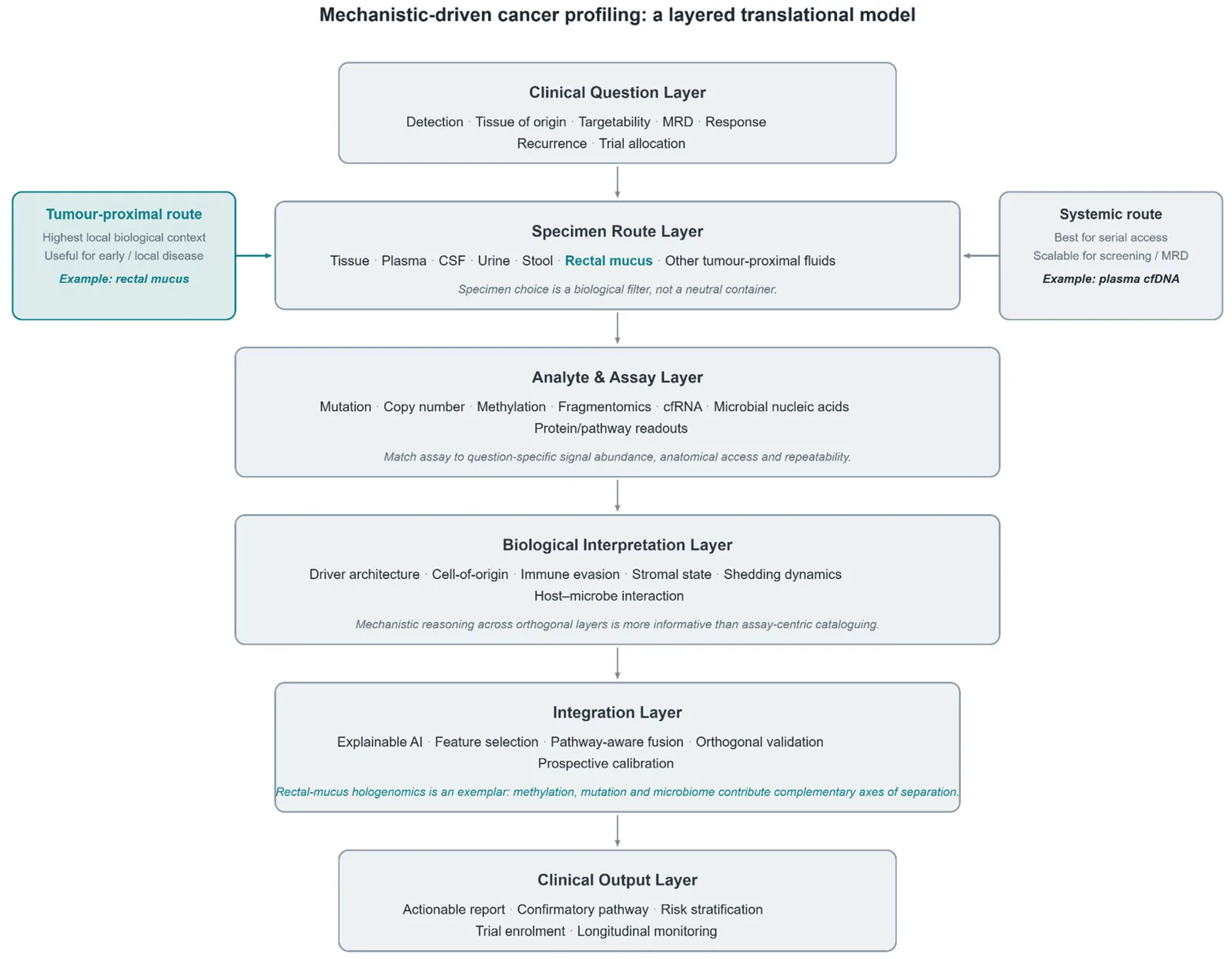
Mechanism-driven cancer profiling as a layered translational model. Clinical questions define specimen appropriateness; specimens filter which analytes are recoverable; analytes map to biological layers; and integrated interpretation informs outputs such as detection, tissue-of-origin inference, targetability, minimal residual disease, and pathway allocation. The rectal-mucus hologenomic study is included as an example of tumour-proximal specimen sampling that jointly captures host mutation, methylation, and microbial signals from a clinically feasible outpatient workflow.

This review proposes that mechanism-driven cancer profiling should become the organising principle for translation to diagnostic applications (Figure 1). By “mechanism-driven,” we mean that biomarker panels should be built to represent causal or functionally linked disease processes across multiple modalities. Targeted integration of biological regulation, such as genetic oncogenic drivers, hallmarks of immune evasion, metabolic adaptation, cell plasticity and composition, host/tumour-microbial interactions, and spatial heterogeneity, facilitate a stepwise improvement in cancer detection and management, rather than simply maximising classifier performance on narrow analyte datasets [4,12,13,15,16]. This is especially important in pre-malignant or early stage disease detection, where the availability of individual analytes is often sparse, the pathological burden is low and the clinically meaningful event does not exist in a binary diagnostic state [1,6,17]. For example, in CRC, which is increasing across the developed world and in younger populations [18], identification of significant pre-malignant lesions (adenomas) or earlier stage (Stage I/II) CRC leads to increased rates of cure. This is a central tenet of the UK NHS Cancer Plan [19]. However, this has to balance the ability to retrieve a biologically remote sample in a minimally invasive way against the risk to the patient from further investigation based upon poor remote sampling performance.

For clarity, we use the term mechanism-driven diagnostics to describe a translational diagnostic strategy in which the clinical question is used to define, which associated biological process should be assessed to garner likely translatable outputs. The specimen is then selected according to its ability to capture that process and the assay or model is judged not only by discrimination metrics but also by biological plausibility, analytical robustness and downstream clinical utility, as opposed to considering standalone interacting biological networks. A biomarker should be considered mechanistically grounded when it informs direct functional involvement in oncogenesis, demonstrates a reproducible association with a biologically supported pathway, is concordant across orthogonal analytes or specimen types, or demonstrates prospective evidence that the biomarker improves a clinically relevant decision.

## 2. The Limitations of One-Dimensional Biomarker Assessments

This critique is not intended to diminish the value of established single-analyte biomarkers, routine clinical practice depends on many highly useful biomarkers, including conventional blood chemistry, tumour markers, immunohistochemistry, molecular companion diagnostics, and faecal immunochemical testing. Their clinical value derives from analytical validity, reproducibility, pathway integration, and demonstrated utility, even when the full causal biology is incompletely resolved. The limitation addressed here is narrower: a single biomarker should not be assumed to represent the full biology of a heterogeneous cancer state and in many instances cannot account for causality.

In CRC, for example, the canonical “adenoma–carcinoma sequence” is described as sequential pathway disruption, following a distinct pattern and cadence [20].

However, organoid work has shown that mismatch repair (MMR) deficient human colon organoids can acquire mutations across WNT, EGFR, p53, and BMP pathways to recapitulate oncogenic progression, demonstrating the heterogeneity and variability of malignant transformation as a multistage, state-dependent process, that is distributed across multiple biological pathways [21]. Understanding the underlying biological process led to the use of targeted therapy away from the traditional clinical/surgical approaches, which was groundbreaking when presented in 2024 in early-stage CRC [22].

Single-cell and lineage tracing studies reinforce these findings in vivo. A large single-cell atlas tracking the transition and field change of normal colonocytes to an adenoma and ultimately colorectal cancer identified a continuum of epithelial, stromal, and immune state changes, including expansion of stem-like epithelial states, pre-cancer-associated fibroblasts, and later T-cell exhaustion, rather than one definitive biological event [16]. Separate work on precancerous lesion evolution showed a transition from polyclonal precursor lesions to more monoclonal malignant states, suggesting that early lesions can be biologically diverse before converging on clones with the greatest proliferative advantage [23]. These findings highlight the disadvantages of relying on single static biomarkers to represent evolving disease development.

Organoid models, lineage-tracing approaches, single-cell profiling, and spatial methods therefore answer related but non-identical questions. Organoids enable controlled functional perturbation and therapeutic testing, lineage-tracing reconstructs clonal dynamics, single-cell methods define cell-state composition, and spatial approaches preserve tissue architecture and cellular neighbourhoods. Their diagnostic relevance lies in nominating mechanisms and candidate biomarkers that can later be translated into simpler, robust assays appropriate for routine specimens.

A broader view of biological mechanisms add context through dysregulation outside of canonical oncogenic driver genes. For instance, in early colorectal cancer models, SOX17 has been shown to suppress IFNγ sensing and promote differentiation towards immune-evasive LGR5-negative tumour cells with reduced MHC-I expression, linking early cancer progression to immune escape rather than proliferative signalling alone [13]. A classifier limited to common driver mutations would miss such biology, despite its likely relevance to progression, treatment response and recurrence.

The same principles have been implicated in therapeutic target selection. Proteogenomic analysis of colon cancer identified protein and phosphorylation features, as candidate biomarkers for therapeutic intervention not directly interrogatable from genomic analysis alone [9]. Additionally, integrated genome and transcriptome profiling in over 1000 colorectal cancers revealed five prognostic cancer subtypes and multiple survival-associated alterations spanning multiple biological pathways, structural variants, and copy number alterations [14]. Proteogenomic characterisation of primary colorectal cancer and metastatic progression further demonstrates that protein-defined subtypes and metastatic signatures can provide information beyond DNA or RNA alone [10]. Table 1 illustrates current modalities assessed, biological specimens used and how these approaches are translating in precision medicine applications. A practical limitation is that the methods best suited for discovery are not always deployable in the specimens used for diagnosis. Combined genomic and proteomic profiling from the same small or low-concentration sample may be constrained by analyte input, extraction chemistry, dynamic range, protein degradation, nucleic-acid fragmentation and batch effects. Emerging single sample or low-input workflows can reduce this trade-off, but routine translation still requires strict pre-analytical control and orthogonal validation, especially where multiple biological layers are derived from scarce material. Increased dimensionality is valuable only when each added layer contributes stable, reproducible, and clinically interpretable information. Additional layers can also introduce batch sensitivity, missingness, increased costs, reduced calibration and overfitting, if the dataset size, pre-analytical standardisation, and validation strategy are insufficient.

## 3. Specimen Choice Is a Mechanistic Variable, That Affects Application and Utility

A second major translational consideration is biospecimen selection. In practical terms, specimen choice determines what biological processes can be analysed and how representative they are of the source pathology. Specimen awareness should be separated into several distinct factors: biological proximity to the lesion, analyte recoverability, pre-analytical robustness, clinical accessibility and acceptability, reproducibility and downstream pathway value. A specimen may be biologically proximal but difficult to standardise, highly acceptable but biologically remote, or analytically rich but impractical to collect. The relative weighting of these factors should depend on the intended use case.

In metastatic non-small-cell lung cancer (NSCLC), plasma based Next Generation Sequencing (NGS) provided faster result generation than tissue biopsies, showed very high concordance for guideline recommended biomarkers assessed, and identified actionable alterations before tissue analysis [24]. However, the same study also acknowledged the limitations of liquid biopsy, including false negatives based on limited relevant biological material and confounders, such as clonal haematopoiesis, introducing age-related mutations into the cfDNA fraction [6,7,8,24].

Plasma is well suited to systemic tumour shedding, but many tumours release little detectable circulating tumour DNA (ctDNA), particularly in the early stages of disease or are blocked by anatomical barriers [1,6]. In glioma, a prospective multicentre study found ctDNA in cerebrospinal fluid (CSF) in 59% of assessable patients with 84% tumour-CSF mutational concordance, whereas plasma detection reached only 14%, explicitly demonstrating the impact of anatomical relevant sampling [25]. Biospecimen dependence is equally visible in established or emerging non-blood platforms.

Urine, saliva, mucus, stool, and device-enabled minimally invasive sampling have extended this principle. A recent study in JAMA Oncology, detailed the development and external validation of an 18-gene urine test for high-grade prostate cancer, with the resultant model demonstrating a 95% sensitivity for high-grade disease [26]. The translation of these results would make a meaningful reduction in unnecessary invasive biopsies relative to the current more established biomarkers and testing procedures. Cytosponge™-TFF3 (Medtronic, Minneapolis, MN, USA), a non-endoscopic oesophageal cell-sampling test, increased Barrett’s oesophagus detection by approximately ten-fold in primary care settings, whilst also supporting the procedure as a cost-effective solution, based on trial modelling [27,28]. These examples highlight how anatomically rationalised sampling has diverse scope and enhanced potential for clinical translation and precision medicine applications.

**Figure 2:**
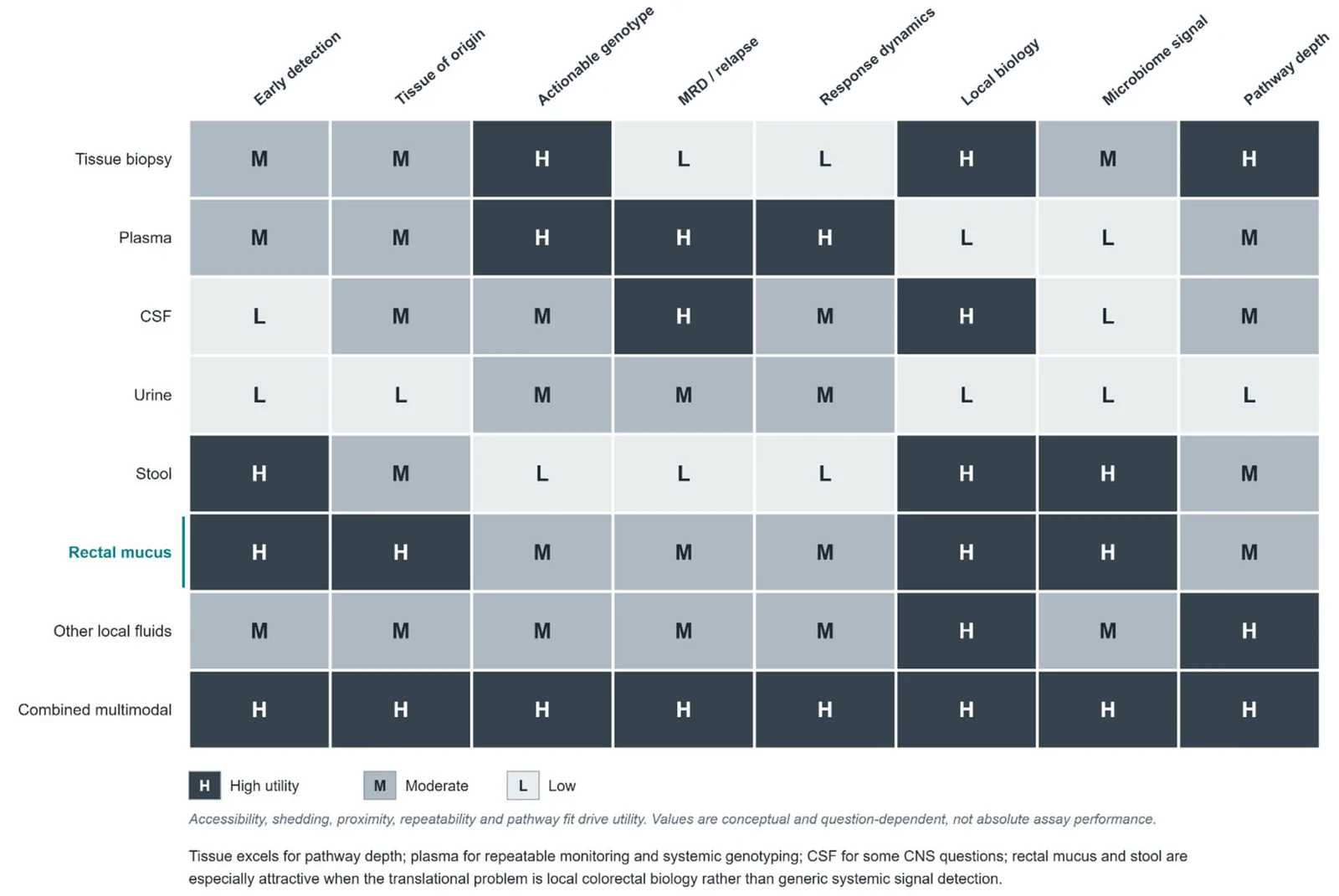
Specimen-by-question heatmap showing the non-equivalence of biospecimens across translational questions. “H”, “M”, and “L” indicate relative expected utility, not absolute assay performance. The figure foregrounds anatomical proximity, tumour shedding biology, repeatability, and pathway fit as the dominant determinants of specimen suitability.

Accordingly, we highlight a series of simple and rationale queries to inform diagnostic assay development: Where is the most informative disease signal most likely to be recovered? Can a representative biospecimen be collected in a patient acceptable manner? Do the analytes maintain suitable stability for analysis (Table 2)? For central nervous system (CNS) tumours, that may be cerebrospinal fluid (CSF) [25]. For urothelial or prostate cancer, urine may carry decisive information [26]. For colorectal cancer and gut–host interactions, rectal mucus sampling may better reflect relevant biology than plasma or stool (Figure 2). A recent *Nature Communications* study provides a direct example of specimen choice acting as a mechanistic determinant of diagnostic utility. By treating colorectal cancer as a mucosal pathology rather than only a systemic shedding problem, the study recovered disease-relevant host and microbial signals from a single anatomically proximal specimen. Interestingly, several significant bacterial indicators identified, are not previously described in stool-based studies [29].

These investigational results highlight rectal mucus as a promising tool for clinical translation to complement the current CRC diagnostic and triage methods, such as colonoscopy, histopathology, stool-based testing, and, in selected settings, blood-based assays.

## 4. Host Microbial Interactions: Homeostatic Functions Versus Mechanistic Cancer Drivers

Microbial communities contribute to mucosal homeostasis, metabolism and immune regulation, but a reproducible association with cancer does not by itself, establish a causal role in oncogenesis [3,4,5,12,15,30,31,32]. In CRC, pooled analyses have identified microbial features that recur across cohorts, including taxa of oral origin that are enriched in the colorectal tumour-associated microbiome [33]. Pan-cancer analysis of metastatic tumours has also linked microbial composition with organ tropism, tumour hypoxia, neutrophil infiltration and patterns of resistance to immune-checkpoint therapy; one reported association was between Fusobacterium and immune-checkpoint blockade (ICB) resistance in lung cancer [34]. These findings support further investigation of tumour-microbe interactions, while retaining a distinction between correlation, diagnostic utility and direct mechanistic involvement.

Interpretation is particularly difficult in low-biomass specimens. Blood and tissue from ordinarily sterile compartments may contain very few microbial reads, making results vulnerable to reagent or laboratory contamination, batch effects and misclassification of host reads [35]. Gihawi et al. reanalysed the data underlying a now-retracted study that had reported cancer type classification from blood-based microbial signatures and did not reproduce the claimed cancer-specific signals for most tumour types [36]. This reanalysis does not invalidate microbial biomarkers as a translational tool but demonstrates that evidence from low-biomass compartments requires stringent controls, contamination-aware processing and independent replication. Signals obtained from stool, saliva or mucosal specimens are not subject to the same assumption of sterility, although they remain susceptible to demographic variation, environmental differences and contamination.

Despite these limitations, microbial features remain desirable for diagnostic development because they can be recovered from tissue, stool, saliva and, in selected settings, blood-derived nucleic acids, whilst complementing host-derived analytes [3,4,5,12,15,30,31,34,37]. A faecal microbiota signature for pancreatic cancer showed high disease specificity which improved further when combined with CA19-9 [30]. In colorectal neoplasia, studies integrating stool microbiota with proteomic, amino-acid and plasma or faecal metabolomic data have distinguished adenomas and CRC by identifying candidate signatures across the adenoma-to-carcinoma sequence [4,5,12,15]. Hologenomic analysis of rectal mucus similarly combined microbial features with host mutation and methylation signals to support detection of adenomatous polyps and CRC from a minimally invasive, tumour-proximal specimen [29]. Collectively, these studies suggest that microbial information is likely to be most useful when it complements host biology rather than being treated as a standalone replacement.

Clinical translation nevertheless requires evidence beyond discrimination in exploratory discovery cohorts. Microbial composition varies across ethnicity and geographic groups [38], while public microbiome datasets are disproportionately derived from highly developed countries [39]. Increasing cohort size by combining existing datasets may therefore introduce systematic population and technical bias unless collection, processing and metadata are sufficiently harmonised. Candidate microbiome biomarkers should be evaluated in representative populations, using independent cohorts and predefined analytical controls. This requirement is particularly important when the proposed biomarker has an uncertain mechanistic basis, because analytical validity, biological plausibility and clinical utility must then be established separately [40,41,42].

Mechanistic support may strengthen translation where microbial features can be linked to tumour-microbe interactions, carcinogenic metabolism or host immune signalling [3,4,30]. Multiomic studies further indicate that microbial signals can be interpreted alongside host transcription, methylation, metabolism and immune function [4,5,12,15,29,31]. However, microbial profiles are context-dependent and may be altered by antibiotic exposure, diet, geography, ethnicity, comorbidities, inflammation and recent treatment. Microbiome-derived biomarkers should therefore be judged by reproducibility, contamination control, biological plausibility, external validation and additive value to host-based biomarkers. Within a mechanism-driven, specimen-aware framework, their most credible role is as complementary evidence of the local tumour ecosystem rather than as an independent proxy for cancer biology.

## 5. Multiomics and Multimodal Integration

If specimen choice determines which signal can be seen, multiomics determines how much of the underlying mechanism can be reconstructed. Integrated genome and transcriptome analysis of CRC has identified prognostic driver mutations, co-dependent mutation patterns, mutational process/pathway signatures, and transcriptional led subtyping that interlink tumour genetics and expression with patient outcome [14]. Proteogenomic studies have added an additional layer by identifying post-transcriptional dysregulation, identifying subtypes defined by protein expression and metastatic signatures that are otherwise incompletely predicted [9,10].

**Figure 3:**
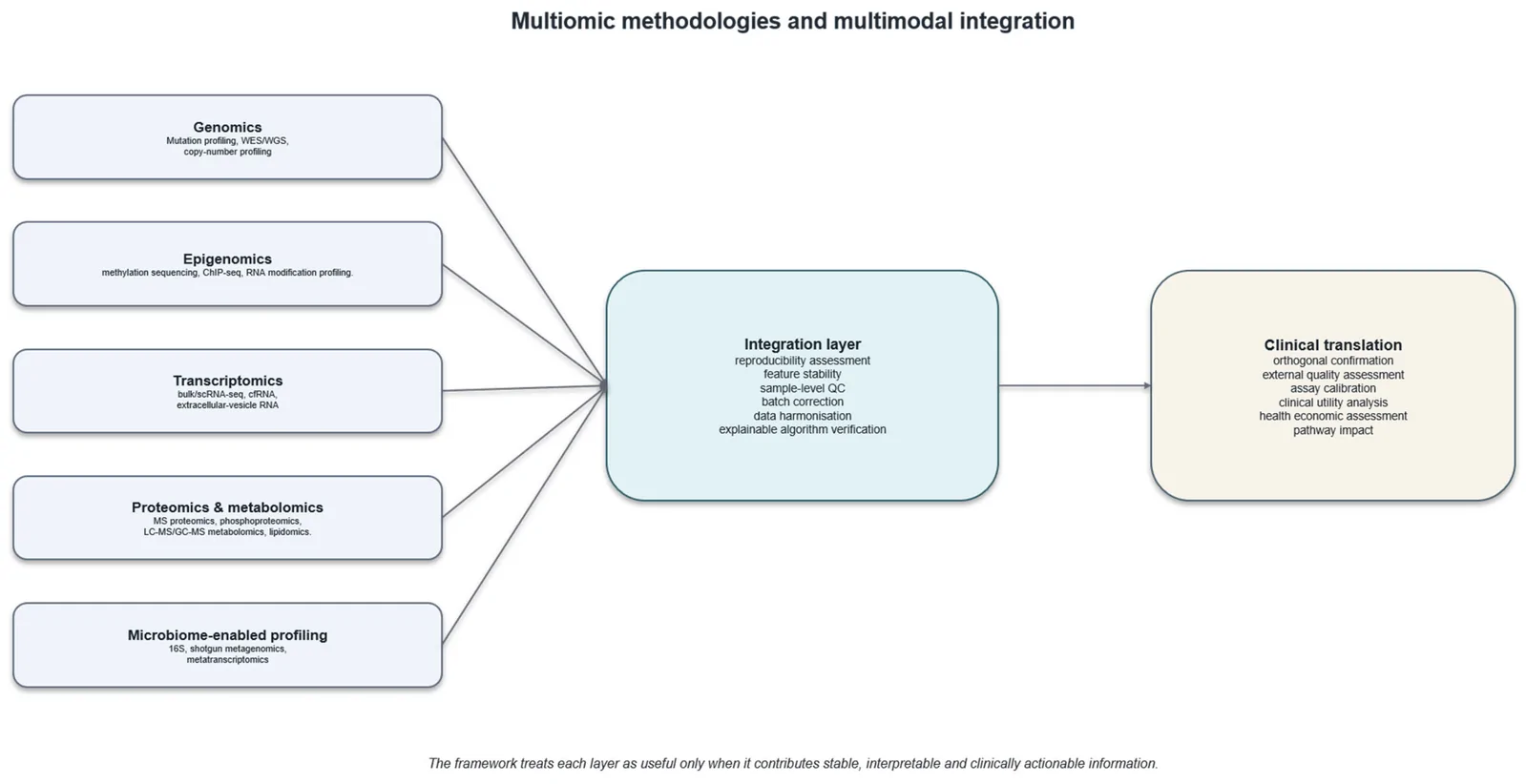
Principal technique classes used in multi-omics and multimodal cancer profiling. The figure details techniques used for analyte data generation, considerations for integration, and validation for clinical translation, including sample-level quality control, batch correction, feature stability, external quality assessment and formalised clinical utility assessment.

Many clinically actionable pathologies are emergent rather than singular, with copy number alterations, transcription factor activity, phosphorylation signalling transduction, cell lineage plasticity, hypoxia and immune infiltration/activation, which can each modulate prognosis, progression and drug sensitivity independently of a single ‘driver’ [9,10,13,14,16]. Integrated studies of CRC development, progression and metastasis show enrichment of additional structural variants and copy number changes in metastatic lesions together with proteomic plasticity and functional signatures such as hypoxia, stemness, and immune profiles [9,10,14]. These observations support a shift from single analyte studies to multi-layered mechanistic classification (Figure 3).

Single-cell and spatial analysis further refine this model by resolving the source of indicative signals, be that tumour cells, fibroblasts, endothelial cells, myeloid cells, or exhausted lymphocytes, and whether they are locally restricted or broadly distributed [13,16]. This distinction is crucial in diagnostic development and biomarker translation because an assay’s robustness can be limited if measured biology is diluted without means of normalisation, spatially concealed, or mis-attributed to an erroneous biological compartment. Advances in AI are increasingly being used to integrate such multimodal datasets for target discovery, biomarker selection, patient stratification, and response prediction, but the model’s performance is only meaningful when the underlying data generated and attribution represent the relevant biological context [17].

Multimodal integration also creates data-governance and infrastructure requirements. Depending on the analytes used, datasets may require harmonised metadata, increased storage security, versioned controlled preprocessing pipelines, data dictionaries, further external quality assessment, heightened sharing controls, and further audit trails that preserve sample traceability. Responsibility for standardisation and protection will vary by healthcare system, sponsor, laboratory network, and regulator, but the minimum requirement is that diagnostic claims remain traceable to validated input data, locked analytical pipelines and predefined clinical definitions.

## 6. Translational Applications Across the Cancer Spectrum

At the screening and early detection end of the pathway, cfDNA methylation, RNA analysis and fragmentomics are the most mature broadly targeted strategies (Table 3) [11,43,44,45,46,47,48,49,50]. In the Circulating Cell-free Genomic Atlas (CCGA) programme, a targeted methylation multi-cancer early detection (MCED) assay achieved very high specificity, detected more than 50 types of cancer, and correctly predicted cancer signal origin in many positive cases, although sensitivity varied substantially by cancer stage and type [11,44]. Jamshidi et al. subsequently showed that whole-genome methylation was the strongest cfDNA feature class among several approaches evaluated for MCED [43]. More recent multi-dimensional fragmentomic work reported 87.4% sensitivity and 97.8% specificity in an independent validation cohort, with 82.4% tissue of origin accuracy, although prospective performance in asymptomatic individuals was more modest at 53.5% sensitivity with 98.1% specificity [47]. Related early-detection platforms, including blood-based machine learning assays, stool RNA testing, and multimodal cfDNA TAPS, further illustrate the expanding range of methylation, RNA, and fragment-structure approaches [48,49,50]. These data are promising but also a reminder that the performance in case control studies is not interchangeable with performance in real-world screening [45,46,51,52].

The current regulatory and evidence landscape support a more cautious approach. The FDA’s advisory process for MCED tests has explicitly centred on study design and the balance of probable benefits and risks [51]. In parallel, National Cancer Institute’s Vanguard Study (NCT 06995898) is enrolling people without cancer to evaluate how MCED tests perform as screening tools, how positive and negative results affect decisions, and ultimately whether such tests present health economic benefit [52]. It is essential that translational research is not focused on classification signals alone and that downstream clinical utility and diagnostic pathway impact are integrated into detection panel selection, clinical study design and test development.

Colorectal cancer provides a useful contrast because translation is already more established. The FDA-approved Shield^TM^ blood test (Guardant Healthcare, Palo Alto, CA, USA) is indicated for average-risk adults aged 45 years and older [53]. According to the FDA summary and the NCI commentary of the ECLIPSE Study (NCT04136002), Shield^TM^ detected colorectal cancer in ~83% of cases, with ~90% specificity, but sensitivity for advanced precancerous lesions was significantly reduced to ~13% [53,54,55]. A later large prospective PREEMPT CRC (NCT04369053) study similarly reported acceptable cancer detection with limited advanced precancerous lesion performance, underscoring the fundamental trade-off in blood-based screening; convenience may improve uptake, but reduced precancerous lesion detection can limit prevention potential relative to colonoscopy or the best stool-based approaches [56]. Those with recent experience in the UK National Health Service (NHS) of the Galleri^®^ blood test (Grail, Menlo Park, CA, USA) had concerns about the interim data in 2024 [57], with the recently published results at ASCO 2026 reporting a failure to meet the primary endpoint of the trial—a reduction in 12 specified Stage III and IV cancers. This has led to further questions as to the cost of implementation within the NHS and concerns about over-investigation of false positive results [58].

This compromise is demonstrated clearly in stool testing. The BLUE-C trial (NCT04144738), a next-generation multitarget stool DNA test showed superior sensitivity for CRC when compared to blood-based methods, including advanced precancerous lesions with a 43.4% sensitivity, albeit with lower specificity [59]. Although the most advanced stool-based tests demonstrate superior diagnostic accuracy compared with blood-based assays, patient compliance remains substantially lower, at ~70%, versus more than 90% for blood-based testing [54,55,59]. Together, the blood and stool based colorectal studies make a broader point relevant to all translational biomarker development, the most appropriate biomarkers depend on the objective; to maximise uptake, detect invasive disease, detect precursor lesions, minimise false positives, or generate a procedure for internal triage of a healthcare system [49,53,54,55,56,59]. However, neither stool- nor blood-based testing has impacted the number of colonoscopies being performed; indeed, data from the UK NICE-FiT Study, which led to the introduction of quantitative faecal immunochemical testing (qFiT), has led to increased numbers of referrals and further investigation, which contrasts with the stated aim, whilst creating a standard that novel minimally invasive tests have to perform against [60].

In diagnosis and frontline treatment selection, mechanistic specimen matching is already producing practical gains. “Plasma-first” or plasma-concordant sequencing in metastatic NSCLC reduced diagnostic turnaround time and can expand actionable biomarker yield without obvious compromise in outcomes [24], even though CSF outperforms plasma for ctDNA profiling in glioma [25]. Urinary RNA testing can reduce unnecessary prostate biopsies while preserving sensitivity for clinically significant disease [26]. These advancements are further examples of how translation outputs improve when analytes, disease topology, and the clinical requirements are aligned.

These studies and datasets are clear advancements with evident promise, although it remains integral to recognise the fundamental clinical positioning of the assay and the differing thresholds and requirements per application. For instance, screening requires very high specificity, an acceptable confirmatory pathway, and evidence that benefits outweigh false positives and over-investigation. Symptomatic diagnosis prioritises timely triage and safe rule-out performance. Therapy selection requires rapid, actionable genotyping or pathway inference. MRD detection requires high sensitivity at very low disease burden and evidence that earlier molecular detection leads to beneficial intervention. A modality that is useful in one setting may therefore be inappropriate in another.

Table 3 provides examples of emergent technology segregated by approach and stage of translation. For example, Shield has regulatory approval for average-risk CRC screening, whereas multidimensional fragmentomics remains near-translated research that requires prospective pathway validation and direct comparison with the current standard of care.

The value of minimally invasive molecular diagnostics is now well established, with applications spanning cancer detection, treatment selection, disease monitoring, and recurrence surveillance [1,6,7,8]. Among these, MRD assessments have emerged as a major focus, aiming to identify molecular evidence of residual malignant cells following curative-intent therapy [1,7,8]. Most current MRD approaches rely on the detection of ctDNA in plasma, enabling the identification of residual disease months before conventional radiological progression becomes apparent and potentially informing treatment escalation or de-escalation strategies [1,6,7,8]. Despite this promise, important challenges remain. Prospective evidence demonstrating that MRD-guided intervention translates into improved survival outcomes is still limited, raising the possibility that earlier molecular detection may simply advance the timing of relapse recognition without altering the disease course [7,8]. Furthermore, reviews of ctDNA-based MRD monitoring in solid tumours consistently highlight several barriers to clinical implementation, including limited assay sensitivity at very low tumour burdens, variability in ctDNA shedding between tumour types and patients, false positive findings arising from clonal haematopoiesis or technical artefacts and uncertainty regarding the optimal management of patients in whom relapse is detectable only at the molecular level [1,6,7,8].

## 7. Methodological and Implementation Considerations

The credibility of a mechanism-driven diagnostic framework ultimately depends on the rigor of its analytical methodology. For liquid biopsy, pre-analytical handling remains a major determinant of performance, blood collection tubes, processing time, centrifugation, storage conditions, and extraction methods can drastically alter ctDNA yield and background DNA contamination [1,6,61,62,63]. More generally, tumour shedding differs by tumour type, burden, vascularity, stage and anatomical location, making false negatives biologically predictable rather than sporadic [1,6,7,8]. Clonal haematopoiesis further complicates interpretation of plasma DNA, especially when low allele frequency variants are used in early detection or MRD contexts [6,7,8,24].

For microbiome associated oncology, rigorous contamination mitigation and host-read handling are required, with uncertainty remaining around demographic generalisability and legitimacy of causal linkage, with orthogonal validation being essential [32]. For AI-enabled multimodal classifiers, external validation, generalisability across populations, feature stability, and interpretability are central translational requirements. Recent reviews on AI in translational cancer research stress that machine learning can accelerate target discovery, biomarker identification, patient stratification, and treatment prediction, but it can also amplify dataset bias and obscure mechanism if deployed as a black-box classifier, without robust authentication [17].

Healthcare system implementation requires another layer of robustness. Asymptomatic screening tests in particular need pathways for confirmatory diagnostics, management of incidental or false-positive signals, interval retesting, reimbursement, and equitable access [51,52]. The NCI Vanguard design is a useful model because it explicitly studies not only assay output, but decision making after normal and abnormal results [52]. Similarly, translational biomarker studies are increasingly judged not only by AUC or sensitivity/specificity, but also by whether they improve the full diagnostic pathway [51,52].

**Figure 4:**
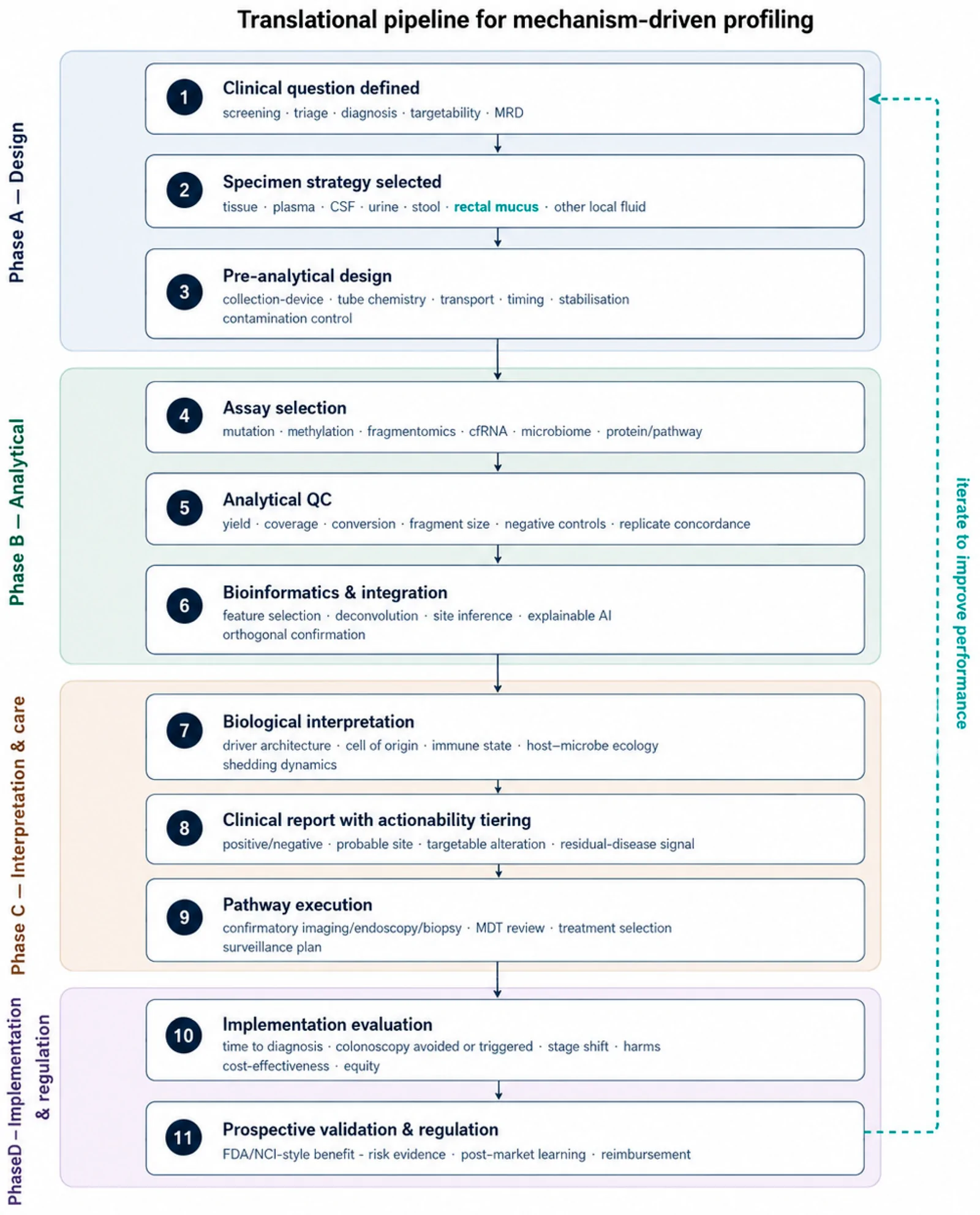
Translational pipeline for mechanism-driven cancer profiling, from clinical question and specimen choice to regulated implementation. The flow emphasises pre-analytics, analytical validity, clinical validity, integrated interpretation, care-pathway consequences, and prospective utility studies. This framework is consistent with FDA and NCI framing for early-detection tests.

Future studies should examine stage shift, time to diagnosis, unnecessary procedures avoided or triggered, treatment selection accuracy, recurrence detection that changes outcomes, patient-reported acceptability, equity of access, cost-effectiveness, reimbursement feasibility, and impact from false positive or false negative classifications (Figure 4). The collation of these findings strengthen health economic and outcomes research enabling representative net benefit analysis.

## 8. Future Directions

The next generation of cancer profiling should be defined less by the expansion of biomarker candidates and more by the construction of mechanistically coherent composite signatures [1,6,12,15,17,29,47]. In practice, panels that deliberately combine tumour intrinsic biomarkers with host response and microbial communities facilitate a contextual understanding of the fundamental biology within pathogenesis. Moreover, further considerations should be placed on the sampling strategies selected and how the results generated are supported by anatomical and biological credibility, while model training and analytics, should be applied to a clinically relevant question, to ensure the highest utility is achieved.

Equally, progress in translation will depend on moving multiomic analysis from retrospective explanation to prospective intervention. Biomarker validation studies that measure stage shift, treatment selection efficiency, time to diagnosis, over-treatment, under-treatment, patient acceptability, and survival, will allow further applications beyond analytical validity [51,52]. The strongest future candidates are likely to be those that integrate high signal analytes, such as methylation or fragmentomics, with a broader understanding of regulated pathway activity and its linkage to the microenvironment, in a tissue-relevant matrix [4,5,9,10,11,12,15,29,30,31,43,44,47,50].

The general principles that are likely to remain stable are that specimens are not biologically interchangeable, clinical utility is pathway-dependent, and additional molecular layers require justification. Specific assays, effect sizes, and early performance estimates are likely to change as technologies and datasets mature, findings are recapitulated/reinterpreted, and prospective trials are reported.

## 9. Conclusions

Cancer translation is entering a new phase where context matters as much as detection. This reviews central findings are that minimally-invasive testing should not be judged solely by whether it can find cancer, but by whether it can recover enough biologically relevant information to guide meaningful clinical action [1,6,7,8,29]. One-dimensional biomarkers remain useful in selected settings, especially when the biology is dominated by a single highly informative signal. In the context of cancer screening, cancer subtyping, personalised therapy, MRD, and longitudinal monitoring, the field is moving toward deeper models that combine mechanism, specimen relevance, and multimodal interpretation [9,10,11,17,24,25,47].

The proposed framework should therefore be understood as a contextual rationalistion of approaches taken, not as an argument that maximal complexity is always superior. Rather, it argues that each translated assay should represent the biology most relevant to the clinical decision it is meant to support [6,17,29]. That principle, more than any single technology, is likely to define the next era of translational cancer diagnostics.

## Figures and Tables

**Table 1:** Comparison of profiling modalities for mechanism-driven cancer profiling.

Modality	Typical Specimen	What it Captures	Main Translational Strengths	Main Limitations	Best-Fit Use Cases
Targeted mutation profiling	Tissue, plasma, CSF, tumour-proximal fluids	Driver alterations, resistance mutations, tumour-informed tracking	Direct link to targeted therapy. Clinically mature in advanced disease	Low shedding/early disease limited detection. Clonal haematopoiesis in blood	Advanced disease genotyping, resistance, tumour-informed surveillance
DNA methylation signatures	Plasma, tissue, local fluids	Cell-of-origin, epigenetic dysregulation, early transformation	Strong tissue-of-origin signal; useful for early detection/MCED	Early disease limited detection. Pre-analytical sensitivity. Platform based variation.	MCED (multi-cancer early detection), screening adjuncts, subtype inference
Fragmentomics	Plasma	cfDNA fragment size, end motifs, nucleosomal organization	Whole-genome structural signal; complements mutation/methylation	Requires robust computational modelling and harmonised processing	MCED, screening, residual-risk stratification
cfRNA profiling	Plasma, extracellular vesicles, selected local fluids	Dynamic transcriptional state, immune signals, host–microbe transcription	Adds biology not evident in DNA alone; can improve site classification	RNA instability. Increased contamination risks.	Detection/classification adjunct, treatment-response biology
Microbiome community and functional analysis	Stool, mucus, tissue, plasma/cfRNA in selected settings	Host–microbe ecology, mucosal disease context	Strong mechanistic relevance in mucosal cancers, that may enrich local signal.	Low-biomass contamination risk, host-depletion and batch correction often required	CRC and other mucosal tumours, mechanistic stratification
Proteogenomics	Tissue, occasionally enriched fluids	Pathway activation, protein abundance, phospho-signalling	Most mechanistically proximate to phenotype and drug response	Tissue-intensive. Analytically complex. Limited routine deployment.	Trial-embedded precision oncology, target discovery, resistance biology.
Integrated multi-omics with AI	Clinical question dependent.	Cross-layer structure across orthogonal signals	Highest potential when layers are complementary and clinically aligned	Overfitting, data sparsity, explainability, external validation burden.	Complex classification, pathway triage, translational biomarker development

Targeted mutation profiling and selected ctDNA assays are currently the most clinically mature, methylation and stool-based tests are clinically available in defined screening contexts and fragmentomics, cfRNA, microbiome-enabled profiling, proteogenomics, and AI-integrated multi-omics remain largely investigational or clinical trial-embedded.

**Table 2:** Specimen-aware framework linking specimen choice to the clinical question.

Clinical Question	Preferred Specimen Class	Why This Specimen Is Favoured	Important Confounders	Example from Current Literature
Is there a systemic actionable genotype?	Plasma ± tissue backup	Fast, minimally invasive, repeatable, good in higher-shedding disease	Clonal haematopoiesis. False negatives in low-shedding disease	Plasma-first NSCLC with rapid turnaround and high biomarker concordance
Is a CNS tumour molecularly detectable without repeat brain biopsy?	CSF	Anatomical proximity overcomes low plasma shedding	CSF access logistics. Timing/site of collection.	Glioma prospective study showing 59% CSF detection and 84% tumour concordance
Is there a local colorectal neoplasia signal in a symptomatic pathway?	Rectal mucus or stool-based proximal matrix	Captures shed host and microbial material from mucosal disease	Collection standardisation, translatability across all lesion sites.	Rectal mucus hologenomics in symptomatic triage.
Is the aim multi-cancer screening in asymptomatic populations?	Plasma	Operational scalability and broad systemic reach	Unresolved positives. False negatives in early disease. Follow-up pathway complexity.	MCED studies and NCI Vanguard
Is the goal deep pathway dissection or resistance biology?	Tissue ± plasma adjunct	Most information-rich for spatial and proteogenomic context	Invasiveness, sampling bias, repeatability.	CRC genome–transcriptome and single-cell transformation studies.

**Table 3:** Examples of clinically translated or near translation next generation diagnostic methods.

Example	Biomarker Logic	Specimen	Stage of Translation	Key Translational Message
Shield for CRC screening	Plasma cfDNA mutation + methylation + fragmentation	Blood	FDA-approved for average-risk CRC screening in adults ≥45 years.	Blood-based screening can broaden uptake, but low early/precancer sensitivity. preserves a major role for colonoscopy
Galleri/targeted methylation MCED methods	Shared cancer signal + cancer signal origin prediction from cfDNA methylation	Blood	Clinically available in some settings, prospective pathway trials ongoing.	High specificity and tissue-of-origin inference are attractive, but pathway benefit still requires prospective evaluation.
Multidimensional fragmentomics MCED	Whole-genome cfDNA fragment features plus genetic signals	Blood	Near-translated research	Fragmentomics materially strengthens early-detection architectures.
Plasma-first genotyping in metastatic NSCLC	Actionable ctDNA alterations	Blood	Real-world implementation	A “liquid-first” workflow can accelerate time to biomarker result and treatment selection in advanced disease.
CSF ctDNA for glioma	Tumour-concordant compartmental ctDNA	CSF	Investigational but clinically plausible	Compartment-proximal fluid can outperform plasma in anatomically shielded tumours.
Rectal-mucus hologenomics	Integrated host mutation, methylation, and microbiome features	Rectal mucus	Near-translated, prospective pathway development continuing.	Local mucus sampling is clinically practical and mechanistically richer than blood for assessed bowel disorders.

## Data Availability

The original contributions presented in this study are included in the article. Further inquiries can be directed to the corresponding author.
